# CRISPR/Cas13-Based Platforms for a Potential Next-Generation Diagnosis of Colorectal Cancer through Exosomes Micro-RNA Detection: A Review

**DOI:** 10.3390/cancers13184640

**Published:** 2021-09-16

**Authors:** Benjamín Durán-Vinet, Karla Araya-Castro, Juan Calderón, Luis Vergara, Helga Weber, Javier Retamales, Paulina Araya-Castro, Pamela Leal-Rojas

**Affiliations:** 1 Scientific and Technological Bioresource Nucleus (BIOREN-UFRO), Universidad de La Frontera, Temuco 4780000, Chile; b.duran01@ufromail.cl (B.D.-V.); karla.araya@ufrontera.cl (K.A.-C.); helga.weber@ufrontera.cl (H.W.); 2 Center of Excellence in Translational Medicine (CEMT), Biomedicine and Translational Research Laboratory, Universidad de La Frontera, Temuco 4780000, Chile; l.vergara03@ufromail.cl; 3 Innovation and Entrepreneurship Institute (iDEAUFRO), Universidad de La Frontera, Temuco 4780000, Chile; 4 Center for Genetics and Genomics, School of Medicine, Institute of Science and Innovation in Medicine (ICIM), Clínica Alemana, Universidad del Desarrollo, Santiago 8320000, Chile; juancalderon@udd.cl; 5 Doctoral Program in Cell and Applied Molecular Biology, Universidad de La Frontera, Temuco 4780000, Chile; 6 Chilean Cooperative Group for Oncologic Research (GOCCHI), Santiago 8320000, Chile; Jretamales@gocchi.org; 7 School of Medicine, Clínica Alemana, Universidad del Desarrollo, Santiago 8320000, Chile; paulinaaraya@udd.cl; 8 Department of Agricultural Sciences and Natural Resources, Faculty of Agricultural and Forestry Science, Universidad de La Frontera, Temuco 4780000, Chile

**Keywords:** CRC, miRNA, exosomes, CRISPR/Cas systems, molecular diagnosis

## Abstract

**Simple Summary:**

Colorectal cancer is one of the most prevalent cancers, whereas a significant number of cases are diagnosed in late cancer stages, and survival rates drop dramatically. Micro-RNAs (miRNAs) from cancer-derived exosomes have shown promising diagnosis potential. Our review aims to present CRISPR/Cas-based molecular platforms as an inexpensive, swift, and robust detection tool of cancer-derived exosome micro-RNAs to streamline future applications based on the novel CRISPR/Cas-based platforms to achieve early CRC diagnosis.

**Abstract:**

Colorectal cancer (CRC) is the third most prevalent cancer with the second highest mortality rate worldwide. CRC is a heterogenous disease with multiple risk factors associated, including obesity, smoking, and use of alcohol. Of total CRC cases, 60% are diagnosed in late stages, where survival can drop to about 10%. CRC screening programs are based primarily on colonoscopy, yet this approach is invasive and has low patient adherence. Therefore, there is a strong incentive for developing molecular-based methods that are minimally invasive and have higher patient adherence. Recent reports have highlighted the importance of extracellular vesicles (EVs), specifically exosomes, as intercellular communication vehicles with a broad cargo, including micro-RNAs (miRNAs). These have been syndicated as robust candidates for diagnosis, primarily for their known activities in cancer cells, including immunoevasion, tumor progression, and angiogenesis, whereas miRNAs are dysregulated by cancer cells and delivered by cancer-derived exosomes (CEx). Quantitative polymerase chain reaction (qPCR) has shown good results detecting specific cancer-derived exosome micro-RNAs (CEx-miRNAs) associated with CRC, but qPCR also has several challenges, including portability and sensitivity/specificity issues regarding experiment design and sample quality. CRISPR/Cas-based platforms have been presented as cost-effective, ultrasensitive, specific, and robust clinical detection tools in the presence of potential inhibitors and capable of delivering quantitative and qualitative real-time data for enhanced decision-making to healthcare teams. Thereby, CRISPR/Cas13-based technologies have become a potential strategy for early CRC diagnosis detecting CEx-miRNAs. Moreover, CRISPR/Cas13-based platforms’ ease of use, scalability, and portability also showcase them as a potential point-of-care (POC) technology for CRC early diagnosis. This study presents two potential CRISPR/Cas13-based methodologies with a proposed panel consisting of four CEx-miRNAs, including miR-126, miR-1290, miR-23a, and miR-940, to streamline novel applications which may deliver a potential early diagnosis and prognosis of CRC.

## 1. Introduction

Colorectal cancer (CRC) has gained significant relevance during the last five years due to its increasing incidence and mortality. The International Agency for Research on Cancer and World Health Organization referred to CRC as the third most prevalent cancer with the second highest mortality rate worldwide, respectively [1,2]. Furthermore, in 2018, there were 881,000 reported deaths linked to CRC, and new cases may increase up to nearly 2.5 million by 2035 [3,4]. CRC is a heterogeneous disease, mainly developing malignant tumors on the inner colon walls and forming polyps in the rectum. In concordance with the severity of the disease, abnormal growths in the colon can be benign, non-cancerous or malignant [5]. CRC can become malignant over time when a polyp grows out of the inner wall of the colon and rectum, leading to a significant metastasis primarily affecting the liver and, less often, the lungs, bones, spinal cord, and brain [6].

CRC development is associated with several individual conditions, including age, environmental toxin exposure, genetics, alcohol consumption, and diet type. However, the exact mechanisms that trigger CRC are yet unknown [7]. CRC is usually prevented with regular screenings and exercise, while therapeutic regimen measures are comprised of surgery, chemotherapy, and radiation therapy [2]. Unfortunately, there are two significant issues with treatment procedures: (1) all CRC treatment protocols are heavily linked to severe patient toxicities and non-compliance [8], and (2) some cancer cells have shown significant resistance to most widely used treatment procedures such as chemotherapy [9]. Moreover, even with increasing efforts of early screening programs, a significant amount of CRC cases are diagnosed at an advanced CRC stage, often metastases, resulting in patient death [9].

Molecular methods based on cell-free cancer-derived extracellular vesicles, including cancer-derived exosomes micro-RNAs (CEx-miRNAs) which are circulating in the blood, have taken relevance for CRC monitoring and early diagnosis [10,11,12]. miRNAs are naturally stable, actively released and have shown good discerning ability with 76% sensitivity and 76% specificity [13] with numerous potential candidates such as miR-21, miR-23a, miR-1246, and miR-92a [14,15,16]. CEx-miRNAs profiles are primarily evaluated through reverse transcription-quantitative polymerase chain reaction (RT-qPCR) [11,15,16,17,18]. However, RT-qPCR has specific challenges, including the limit of detection and limited throughput, consistency, response-time, and portability [19,20], which may affect reported sensitivity, specificity, and turnaround times, ultimately making clinical care decision-making difficult.

In the last four years, several clinical reports have presented CRISPR/Cas (clustered regularly interspaced short palindromic repeats/CRISPR-associated proteins) as a novel nucleic acids detection method [21,22,23,24,25,26,27,28,29,30,31,32]. Several studies harness these natural molecular beacon features with ssDNA/ssRNA-based fluorescent-quencher reporters (FQR) to establish ultrasensitive, inexpensive, multiplexed, and swift molecular detection platforms [21,22,25,31]. Thus, showcasing promising tools for worldwide early diagnosis efforts for CRC.

This review presents the potential of CRISPR/Cas-based platforms that represent a significant opportunity for next-generation, early point-of-care (POC) detection of CRC via detecting its CEx-miRNAs. To achieve this, we briefly summarize CRC relevance, current CRC diagnosis tools, their challenges, and current clinical CRC biomarkers. Therein, we revise extracellular vesicles and their cargo importance, focusing on their miRNAs and diagnosis significance to ultimately engage over CRISPR/Cas systems and their reported diagnosis platforms to propose potential methodologies that may streamline future CRISPR clinical applications for CRC early diagnosis.

## 2. CRC Relevance, Risk Factors, and Key Stages for Diagnostic Survival

**Figure 1:**
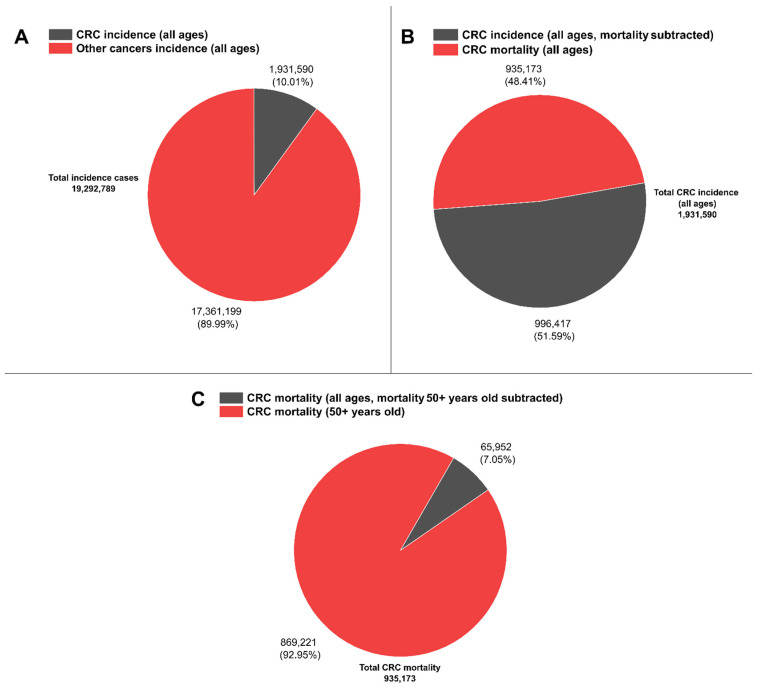
CRC worldwide representative statistics. (**A**) CRC incidence (all ages) vs. other cancer types incidences (all ages). (**B**) CRC incidence (all ages, mortality subtracted) vs. CRC mortality (all ages). (**C**) CRC mortality (all ages, mortality 50+ years old subtracted) vs. CRC mortality (50+ years old). Data are available at [35].

Recent reports on CRC have highlighted that among all cancer cases, 10% correspond to CRC [33], while other reports have denominated CRC “an epidemic” [34]. Moreover, within this 10% of CRC incidence for both sexes (1,931,590 cases from a total of 19,292,789 cases), CRC mortality reached about half for both sexes (48.4%, 935,173 deaths) with respect to its incidence. In contrast, more than 90% of reported total CRC mortality is concentrated in the group of 50–85+ year olds (869,221 deaths, see Figure 1) [35], hence highlighting the current need to further increase early diagnosis efforts by deploying novel, cost-effective methods.

Like other diseases, CRC formation is due to multifactorial events comprising two main components contributing to CRC development: genetic, DNA methylation alter-ations, and environmental elements [36]. Genetic-based studies have estimated CRC heritability to be around only 35–40% [33,37,38], showcasing a significant environmental in-fluence on CRC formation and development. Accordingly, considerable data aim for different environmental risk sources, including gut microbiota [33], dietary patterns, obesity, and smoking (further reading can be found in in-depth reviews [39]).

Currently, CRC is represented by five subtypes: adenocarcinomas (representing over 90% of CRCs diagnoses), carcinoid tumors, gastrointestinal stromal tumors, lymphomas, and sarcomas [33,40]. Clinically, CRC staging is based on three criteria: (1) cancer level of expansion in the intestine wall, (2) affection of other nearby structures, and (3) lymph nodes or distant organs being reached. These are described by the American Joint Committee on Cancer (AJCC) [41]. In brief, AJCC classifies cancer in four stages: (0) abnormal cells present that may lead to cancer, (I) cancer cells present but only locally spread not affecting other nearby tissue, (II) cancer cells present and affecting nearby tissue, (III) cancer cells have reached lymph nodes and, (IV) cancer cells have reached distant parts of the body. Moreover, cancer can also be classified using the TNM staging system, whereas T refers to the primary tumor size which has not yet reached lymph nodes (equivalent to stages I and II of the AJCC), N describes cancer cells that have reached one or more lymph nodes (equivalent to stage III of the AJCC), and M describes whether the cancer has metastasized, i.e., the primary tumor has reached other parts of the body (equivalent to stage IV of the AJCC) [41,42,43].

CRC, its stages, short/long-term survivability, and prognosis predictions have been primarily studied and reviewed elsewhere [44,45,46,47]. Furthermore, reports have high-lighted the sudden decrease of five-year survival rates as TNM or AJCC staging increases [41,48], whereas from T to N, the survival rate decreases by almost 20%, from 90.6% to 72.2%, respectively, and from N to M dramatically drops practically 60%, from 72.2% to 14.7%, respectively [43]. Hence, there is a considerable number of initiatives to obtain reliable and specific tools and biomarkers to establish an early diagnosis of CRC [49]; however, early CRC diagnosis is still a challenge since only 40% of CRC cases are diagnosed at stage I [50] either due to lack of compliance or test-related issues (e.g., sensitivity, specificity, false positives and negatives) from current routine procedures including colonoscopy, fecal immunochemical test (FIT), and guaiac-based fecal occult blood test (gFOBT) [13,51,52]. Thereby, molecular diagnosis has risen as a swift and affordable route to obtain an early CRC diagnosis [53], to be followed by suitable treatments, including chemotherapy [54], radiotherapy [55], immunotherapy [56], targeted therapy [57], and other therapies (further reading can be found in [9,58,59]), thus ultimately preventing, to some extent, patients’ deaths and life-quality deterioration.

## 3. Current CRC Diagnosis and Their Challenges: Traditional and Molecular Methods

In terms of diagnosis, survival rates rely directly on the CRC stage at the time of diagnosis; thereby, CRC can be a preventable and treatable disease with current CRC treatments if an early diagnosis is provided [60,61], considerably enhancing medical outcomes with a five-year survival rate to 90% in cases diagnosed early [62]. Moreover, in contrast with other types of cancer, CRC develops and progresses slowly over the years; it can be up to decades before normal colorectal epithelium transform into an adenoma [34,63]. However, despite systematic public awareness campaigns on CRC and early diagnosis efforts, 50% of CRC-diagnosed patients already carry metastasis [64].

Late CRC diagnoses may be explained by numerous factors playing pivotal roles for diagnosis, including that CRC is comprised of a heterogeneous cancer population, known as consensus molecular subtypes of cancer (CSM1 to 4; further in-depth reading in [64,65]). CSM1 to 4 merges up to 27 CRC subtypes, representing four groups with different gene expression profiles between different regions of the tumor and tumor microenvironment (TME) components [66]. Moreover, intra-tumor heterogenicity (ITH) also drives to spatial heterogenicity [67], where CRC fully differentiates into functional cells and immature cancer stem cells inside the same cancer [67,68]. Thus, from a molecular point of view, ITH directly impacts heterogeneous sensitivity to current, established CRC treatments and their prognosis [69,70]; therefore, early molecular diagnosis efforts may also be affected by CRC ITH, further complicating current early diagnosis efforts.

Established traditional and routinary diagnostic tools have shown good overall efficacy in diagnosis, thus decreasing CRC-related mortality (see Table 1). However, several factors can affect traditional methods and their effectiveness, including low efficacy, high costs, lack of accessibility, limitations of test performance, invasiveness, and suboptimal screening compliance [11,71]. Instead, molecular methods, for which detection is based on either specific segment DNA or RNA obtained from significantly less invasive sampling (e.g., blood samples), have proven to be practical tools, with significant efficacy, lower costs, and faster turnarounds (see Table 1) [20,36,72].

Furthermore, although molecular diagnostic methods have significantly improved CRC screening, similarly to traditional diagnostics, these also carry limitations and challenges. The gold standard technique currently in use, qPCR and RT-qPCR, has been reported to have several restraints [19,20,86,87]. Thus, CRISPR/Cas-based diagnosis (CRISPR-Dx) technologies may represent a potential opportunity to further improve current molecular diagnosis efforts due to their ultrasensitive and robust bio-sensing properties, especially when there have been significant advances in CRISPR-Dx technologies in clinical research and clinical applications [88].

## 4. Current Clinical Molecular Biomarkers for CRC

Molecular methods base their detection on molecular biomarkers, defined as specific and characteristic DNA or RNA segments with high value for diagnostic and prognosis assessments, whether because they are mechanistically implied with the phenotype of interest or rather just correlated to it. There have been multiple efforts to characterize novel and reliable molecular biomarkers for CRC associated with heterogeneity and clinical stages (see Table 2).

Although current molecular biomarkers have a good performance, these perform correctly only within a small population of patients at specific CRC stages and with specific molecular characteristics, rendering them insufficient for a wide-range CRC diagnosis [20,94]. Thus, the research community keeps moving forward to develop new and more accessible molecular biomarkers that can be found more abundantly and with a broader diagnosis range, capable of diagnosing most CRC stages despite its heterogeneity, since CRC early diagnosis is critical for survival and is currently in high demand [95,96].

Interestingly, recent reports indicate that some CRC-related bacteria are related to early and late CRC stages, showcasing bacteria as potential CRC molecular biomarkers for early diagnosis [97]. There are also reports showcasing cancer-derived exosomes miRNAs (CEx-miRNAs) and circular RNA (circRNA) as potential biomarkers for CRC diagnosis and prognosis [98,99,100,101].

Current traditional and molecular methods clinically used for CRC diagnosis require specialized technical expertise and equipment, highlighting the necessity for developing more straightforward, robust, cost-effective diagnosis platforms for broader, bigger, and more accessible use (further revision can be found in [20,96,102,103]). In keeping with this premise, CEx-miRNAs alongside CRISPR/Cas-based platforms may represent a potential candidate to solve these issues, either via POC platforms or clinical-based tests.

## 5. Extracellular Vesicles as Potential Molecular Biomarkers for Early Diagnosis

**Figure 2:**
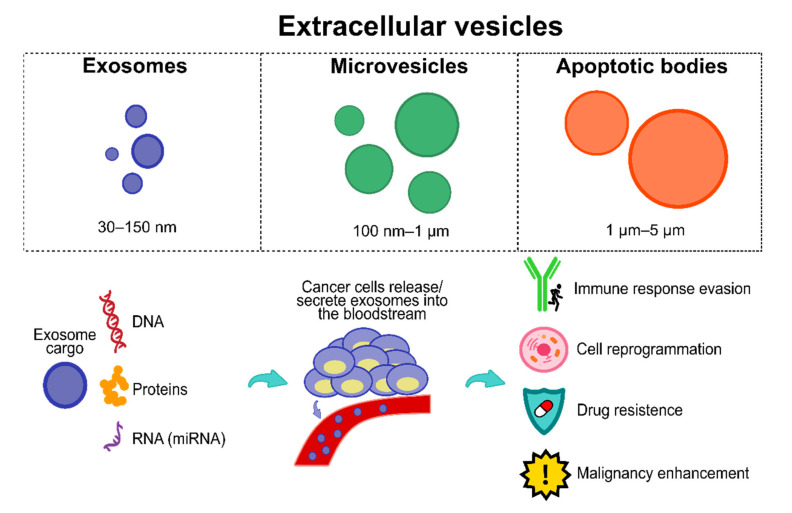
EVs and the importance of exosomes. Exosomes have a plethora of cargo (e.g., proteins, DNA, RNA, micro RNAs, among others). Cancer cells hijack exosomes and their cargo, releasing/secreting them into the bloodstream to obtain several features, including immune response evasion, cell reprogramming, drug resistance, and malignancy enhancement.

Among the numerous biomarkers reported to date, extracellular vesicles (EVs) have been given particular attention by the research community. EVs comprise three vesicle types, including apoptotic bodies, exosomes, and microvesicles (see Figure 2 [104]); they are secreted and released by almost all cells, including cancerous cells.

EVs are physiologically essential since they play a role in two significant functions: (1) cellular waste management and (2) intercellular communication, i.e., EVs are highly stable and efficient cellular communication vehicles with significant cargos including proteins, lipids, metabolites, and nucleic acids (DNA, messenger RNAs, miRNAs, and long non-coding RNAs), with the ultimate purpose of cargo transfer to mediate intercellular physiological and pathological conditions, inducing homeostatic changes on target cells. Structurally, EVs are comprised of a circular phospholipid bi-layer and are unable to divide. This structure is essential because it confers significant stability, resistance to degradation, and longer half-life to its cargo, endowing EVs with an ideal and versatile intercell communication vehicle. Moreover, EVs’ structure allows its cargo to be efficiently delivered into the target cells [105,106,107].

Interestingly, cancer cells hijack and exploit EVs’ signalling network for their bene-fit (see Figure 2), including cell reprogramming towards tumor-promoting intra- and intercellular environments, stimulating cancer development and enhancing survival, angiogenesis, invasion, and metastasis through cargos that support nearby cancer cells to evade immune responses and cell death during all cancer stages [104], which may confer superior diagnostic and prognostic features in comparison to other circulating biomarker types due to their relative abundance, stability, and the array of targets they express [104,105]. It is essential to mention that currently there is no clear consensus on the current release amount of EVs, i.e., studies highlight that cancer cells release significantly more EVs than normal cells, whereas other studies show no significant difference [106]. This issue may be explained due to the lack of standardized protocols and consensus about optimal EV extraction, isolation, and purification from clinical or cell culture samples [106,107]. However, a review study from Choudhry et al. [108] showed how tumor cells significantly increase EV production and release, specifically cancer-derived exosomes (CEx), to induce themselves and nearby cells into hypoxia to enhance the release of higher amounts of CEx, thereby also reaching distant cells to induce hypoxia and ultimately promoting cancer progression. Accordingly, recent reports have also had similar results about the larger amount of CEx released by tumor cells than by normal cells [109,110,111].

Moreover, they play specific roles in CRC malignant progression and responses to therapies [112,113], hence also carrying several potential diagnostical molecules (an in-depth review of EV types, biogenesis, and cargos can be found in [114,115,116,117]). CEx have been shown to alter the origin of the tumor microenvironment and their functional cargo to modulate and support oncogenic mechanisms including angiogenesis, immune modulation, and metastasis, enhancing cancer malignancy features [118,119,120], including chemotherapy and drug resistance (an in-depth review of the role of EVs and CEx can be found in [121]). Furthermore, CEx can be easily obtained from patient biological fluids, including blood, plasma, serum, and urine [122], therefore converting CEx and their cargo into potential biomarkers for CRC surveillance, early diagnosis, and prognosis.

Some attractive benefits from CEx-based diagnostics are (1) less invasive methods, (2) easier patient follow-up of their cancer stage, and (3) easier surveillance of patient cancer relapse; nonetheless, there are also drawbacks, including (1) CEx may be highly heterogeneous due to physiological conditions (e.g., stress), (2) time required and high cost for their analysis, and (3) methods must promise high sensitivity and specificity [16]. Accordingly, CEx-based diagnostics are promising, but several milestones need to be tackled, specially CEx isolation standardization and characterization to obtain cost-effective methods with swift turnaround times.

## 6. CEx-miRNAs for CRC Diagnosis

miRNAs are small non-coding RNAs (18–24 nucleotides) that perform post-transcriptional regulation of mRNAs, mainly playing an inhibiting role when binding to the mRNA 3′ untranslated region, ultimately impeding their translation or leading to degradation [123]. miRNAs regulate several biological processes, including cellular differentiation, proliferation, and apoptosis; thus, they can be easily found in blood samples and are easy to obtain and minimally invasive [123]. Hence, miRNA expression dysregulation can lead to various types of cancer, including CRC [124]. miRNAs are also essential for cancer progression due to reported observations that they play critical roles in regulating cancer signalling mechanisms, enhancing several factors, including tumor growth, angiogenesis, and metastasis [125].

Furthermore, Wang et al. [126] recently demonstrated that CEx-miRNA miR-NA-25 substantially facilitated CRC development and metastasis, pinpointing the importance of miRNAs not only as cancer-promoting molecules but also molecules that carry major diagnosis and prognosis potential for patients (in-depth reading about miRNAs biogenesis, pathways, and their relevance can be found in [123]).

Additionally, several studies have indicated that blood samples have emerged as a reliable source of biomarkers [127,128]. Accordingly, cancer-related miRNAs, either CEx-miRNAs or cancer-derived cell-free miRNAs (cf-miRNAs), from blood represents a promising target for more accessible and non-invasive testing [129,130]. In this regard, blood CEx and miRNA extraction can be easily achieved through available commercial kits, delivering precise and valuable diagnostic and prognostic data [131,132,133].

CRC-related miRNAs can be found in blood in two variations: (1) cf-miRNA and (2) CEx-miRNAs. However, CEx-miRNAs are selectively released by tumour cells, enhancing miRNA specificity and stability compared with cf-miRNAs, which are more vulnerable to degradation [134]. Hence, several studies suggest CEx-miRNAs as a better, easier to obtain, robust, and reliable alternative as a molecular biomarker for cancer diagnosis and prognosis, including CRC [132,135,136]. Accordingly, there are several CEx-miRNAs related to specific CRC staging with a dual biomarker property whereby they can function as diagnosis and prognosis biomarkers (see Table 3). It is pivotal to mention that the best performance results for CRC diagnosis and prognosis are based on CEx-miRNAs panels (e.g., [137,138]), highlighting the importance of multitargeted-based detections to enhance early diagnoses and prognoses for CRC and other cancers.

Moreover, there are also several miRNAs linked to poor performance on early di-agnosis [151], highlighting a current need to characterize CEx-miRNAs and cf-miRNAs further since overall miRNA expression can be altered by several situations, including ITH, treatments, and patient metabolism stresses [145,152]. Thus, further research is needed to simultaneously screen higher numbers of miRNAs candidates to study their fluctuation under different conditions to understand miRNA dysregulation patterns in patients, ultimately enhancing patient diagnosis and prognosis. Accordingly, there is also a need for current and future CRC miRNA biomarkers reports to be in-depth and characterized to be able to discern TNM staging, progression, and their predictive performance (e.g., area under the curve; AUC) as a minimum report standard for a comparable, comprehensive, and accurate diagnosis for CRC and other cancers to achieve swift early diagnostics.

A blood-based miRNA diagnostic for cancers is currently trending and being in-tensely studied [127,136,153,154]. Therefore, the current challenges previously mentioned may soon be addressed and tackled. Moreover, recent reports have aimed to use saliva as a source of cancer-derived miRNAs, obtaining promising results for CRC [155,156]. The use of blood and saliva as a source of biomarkers may help close the gap to achieve reliable and swift early CRC diagnosis and prognosis, providing, in turn, opportunities for novel and innovative technologies such as CRISPR-Dx platforms to aid clinical diagnosis efforts.

## 7. CRISPR/Cas Systems

CRISPR/Cas systems arrange and shape the prokaryotic adaptative immunity and immune memory by acquiring foreign viral genetic material, namely spacers, and using them later to resist invasion [157,158]. Accordingly, CRISPR/Cas classification is made up of Class I (including type I, III, and IV) and Class II (including type II, V, and VI), where both class types currently have 33 characterized subtypes in total [159]. Interestingly, CRISPR/Cas systems are incredibly diverse, which may be explained due to the continuous encounters with different viruses throughout time, driving towards competitive coevolution [160,161].

CRISPR/Cas immune response against viral invasion has three key stages: adaptation, expression, and interference. During the adaptation process, Cas proteins detect their target DNA/RNA (known as protospacer), recognized by a protospacer-adjacent motif (PAM, DNA target) or protospacer flanking site (PFS, RNA target) depending on the Cas effector. Then, Cas proteins bind to the target DNA/RNA to update their immune memory bank, integrate the foreign DNA sequences into the CRISPR array, and acquire a new spacer [162]. In RNA acquisition, a retro-transcription of the target is performed before spacer acquisition and integration into the CRISPR array [163].

The CRISPR array is then expressed as a premature crRNA (pre-crRNA) that will mature and generate a crRNA via either Cas proteins or host factors. This crRNA (often named as gRNA) will then complex with a Cas protein to rise an effector complex and perform the interference stage, which involves the target nucleic acid cognation, binding (tertiary complex), and cleavage, ultimately degrading their target and preventing further exogenous host invasion. Therefore, CRISPR/Cas is a sophisticated RNA-guided adaptative immunity system based on a molecular nucleic acid memory (further in-depth revision of CRISPR/Cas immune acquisition and response process can be found in [157,158,159,160,161,162,163,164]).

Hence, it is important to highlight that Class 1 systems are based on multiple effector modules with several different Cas proteins to provide bacteria and archaea with the adaptative immunity stages. In contrast, Class 2 systems comprise single, multidomain Cas proteins (e.g., Cas9, Cas12, and Cas13) containing all necessary domains and activity to carry the target cleavage, i.e., interference. In some subtypes, some Cas proteins also provide pre-crRNA processing [85], rendering Class 2 systems as straightforward molecular mechanisms to harness and establish molecular detection tools.

Indeed, since the renowned study published by Jinek et al. [165], which showed CRISPR/Cas9 as a new dual-RNA-guided genetic engineering tool capable of precisely performing double-stranded DNA (dsDNA) cleavage, this technology has broadly led to the well-known CRISPR revolution due to its ease of use, versatility, and high efficiency to generate permanent genetic changes on its target, therefore, it was swiftly implemented in several studies on animal and cellular models [157]. Moreover, harnessing CRISPR/Cas9 versatility, it has also been engineered to work as a transcriptional regulator [166], DNA labeler [167], and nucleic acid detector [168]. Thus, the CRISPR/Cas9 toolbox has enabled promising advances, including breast cancer modelling in mice [169], potential CRISPR-based treatments [170], interrogation of mechanisms in ovarian cancer [171], epigenetic control of pancreatic cancer [172], targeted tumor regression [173], and lung cancer miRNA detection [174]. Moreover, CRISPR/Cas technology has recently obtained a considerable milestone in in vivo gene editing to treat transthyretin amyloidosis, achieving the first direct body bloodstream deployment of lipid nanoparticles encapsulating CRISPR/Cas9 mRNA (i.e., Cas9 and gRNA) to safely decline the synthesis of the TTR protein associated with the disease by an average of 87%, whereas conventional methods report up to a 80% TTR synthesis decline [175,176,177]. Indeed, this new CRISPR/Cas landmark registers an important precedent that may be applicable to treat other diseases, including enhanced CRISPR/Cas-based therapies for cancer.

The characterization of the mechanism of action and potential uses of the CRISPR/Cas9 system resulted in the 2020 Chemistry Nobel Prize being awarded to Jennifer Doudna and Emmanuelle Charpentier. However, since its origin, the CRISPR revolution has gone far beyond its use as a genetic engineering tool. New Cas endonucleases have been characterized and have expanded CRISPR/Cas technologies towards novel nucleic acid-based molecular diagnostics, with swift, ultrasensitive, and inexpensive diagnostic platforms, thus branching out to a CRISPR-Dx revolution with versatile next-generation molecular biosensing platforms [29,178].

**Figure 3:**
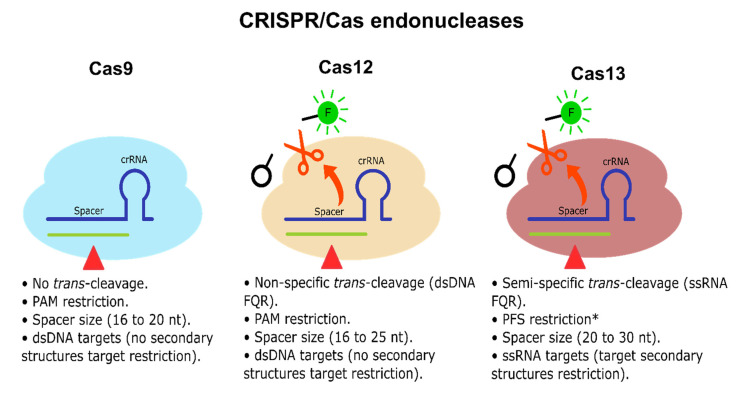
CRISPR/Cas endonucleases. Cas9, Cas12, and Cas13 endonucleases are illustrated. Cas9 and Cas12 cis activity cleaves dsDNA. Cas13 cis activity cleaves linear ssRNA. Cas9 does not exhibit any detectable trans-cleavage activity. Cas12 and Cas13 exhibit trans-collateral activity capable of cleaving non-specific dsDNA/semi-specific ssRNA FQR, respectively. Cas9 and Cas12 do not have target secondary structure restrictions, while Cas13 has target secondary structure restrictions since it only can cleave linear ssRNA [31,182,183]. * Some Cas13 endonucleases exhibit no PFS restrictions [184].

CRISPR-Dx is mainly led by two Class 2 endonucleases, Cas12 and Cas13, which bind and cleave DNA and RNA, respectively. They both have a cis cleavage mechanism of action, i.e., degradation of the main DNA/RNA target. However, they have also been reported to exhibit a target-activated trans collateral cleavage capable of degrading short sequences of dsDNA or single-stranded RNA (ssRNA), respectively, triggered upon target detection and cis cleavage [21,31] (see Figure 3). Thus, these natural Cas12 and Cas13 properties have been quickly harnessed to establish molecular diagnosis tools based on rapid and specific nucleic acid detection mediated mainly through FQR [22,25]. Accordingly, Cas12 trans collateral activity has a cis:trans enzymatical kinetic ratio of 1:1250 per second, showcasing a natural signal amplification of the detection performed [31]. Regarding Cas13, there are no similar studies done on the most widely used type of *Leptotrichia wadei* Cas13 (LwaCas13a); however, inferring from their similar sensitivity (aM vs. zM, with previous amplification), the LwaCas13a cis:trans kinetic ratio might be similar or higher [22]. Accordingly, Shan et al. [179] reported that *Leptotrichia buccalis* Cas13a (LbuCas13a) had shown a cis:trans cleavage ratio of 1:4854 per second with a sensitivity reaching the 1 pM range (no previous amplification). Therefore, Cas12 and Cas13 are not only natural beacon-like reporters, but they also intrinsically amplify the detection signal mediated by their trans-collateral activity. Although Cas9 does not exhibit any trans-collateral activity (see Figure 3), there have been some CRISPR/Cas9-based platforms, including lateral flow detection [180] and fluorescence readouts [181], which display a good detection performance for the CRISPR-Dx toolbox; however, the implementation may have drawbacks due to logical adjustment of Cas9 to transform it into a functional molecular detector, including less cost-effectiveness due to the requirement of antibodies, fluorescent probes, and extra enzymes, which also leads to complications for setup.

Further characterization has been performed on Cas12 and Cas13 subtypes and orthologs, observing that Cas12 trans dsDNA cleavage preference is non-specific [31]. In contrast, Cas13 trans ssRNA cleavage has shown a di-nucleotide motif preference, which varies depending on the Cas13 ortholog [22], allowing multitarget detection, considerably increasing its applicability for molecular diagnosis, especially when several targets must be identified simultaneously within a single sample. Additionally, Cas13 is customizable in terms of portability and one-step reactions maintaining its swiftness, robustness, and sensitivity [22,24]. Therefore, Cas13 and its orthologs (e.g., LwaCas13a, LbuCas13a) represent the best potential candidates among Cas endonucleases to fulfill miRNA-based diagnosis requirements and tackle current challenges for CRC early diagnosis and prognosis, which may also benefit other types of cancer that also have miRNAs as potential diagnosis molecules.

CRISPR/Cas13’s excellent diagnosis capabilities have been proposed and used in several other fields, including SARS-CoV-2 detection [185], food pathogens [186], and environmental biomonitoring and surveillance [187,188]. Interestingly, as CRISPR/Cas technology applicability increases, novel Cas endonucleases are characterized and added to the CRISPR/Cas toolbox [189]. For example, Cas13d has been recently characterized with similar properties to Cas13a, b and c, but with a molecular weight of approximately two-thirds of its predecessors’ molecular weight [184], which may facilitate its expression and reduce overall enzyme production costs for future applications.

Moreover, CRISPR/Cas systems have also been characterized within genomes of huge bacteriophages, namely CRISPR-CasΦ [190], which further expands the CRISPR-Dx toolbox. Accordingly, there is no doubt that considering the bacterial biodiversity (and now viral) there will be several new Cas endonucleases discoveries that may further expand and enhance the current CRISPR/Cas toolbox towards innovative or enhanced functionalities.

## 8. CRISPR/Cas13-Based Platforms as a Potential Candidate for CRC Early Diagnosis and Prognosis

Although most CRISPR/Cas13-based molecular platforms have not yet been broadly used on miRNAs (see Table 4), they portray potential opportunities for CRC and cancer early diagnosis and prognosis from either CEx-miRNAs or cf-miRNAs. CRC has been shown as a worldwide epidemy, and complementary tools currently need to be enhanced to provide primary, secondary, and tertiary prevention to patients. We propose that CRISPR/Cas13-based diagnosis can play a pivotal role in helping secondary prevention measures to meet current needs for CRC early diagnosis and prognosis, whereas early screenings have been shown to help assess and reduce CRC mortality [191]. Accordingly, CRISPR/Cas13-based platforms may also help to provide a better quality of life of the patients with tertiary prevention, playing a role for patients’ prognosis and providing medical teams with data that support the best decision-making process to deliver adequate therapies.

The SHERLOCK platform was the first CRISPR/Cas13 molecular detection tool where Gootenberg et al. [21] showcased CRISPR/Cas technologies as a versatile diagnostic tool and paved the way for the CRISPR-Dx revolution. In the first instance, SHERLOCK only harnessed LwaCas13a nucleic acid detection capabilities, obtaining single-nucleotide resolution with 2 aM sensitivity within 2 h. However, this platform was quickly enhanced the following year, presented as SHERLOCKv2 [22], with several breakthroughs coupling Cas13 reaction with an isothermal amplification method dubbed recombinase polymerase reaction (RPA), obtaining significant enhancements including inexpensive zM sensitivity with linear, quantifiable results, reaching up to aM-zM sensitivity with a cost of USD 0,6 per sample, 30 min one-step runs, four-channel targets detection via multiplexing three Cas13 orthologs and one Cas12 enzyme, and a strip-based test with lateral flow readout, therefore providing an accessible and versatile portable nucleic acid platform.

SHERLOCK diagnosis was then further optimized for clinical samples with a special protocol termed HUDSON [26], enabling SHERLOCK to pair with instrument-free detection directly from patient fluid samples including blood and saliva in less than 2 h, thus proving Cas13 to be a robust endonuclease for direct analysis on unextracted samples. Another improvement of SHERLOCK was then developed by Ackerman et al. [192], where CARMEN-Cas13 represented the first use of SHERLOCK on a larger scale, managing the detection of 169 viruses simultaneously through 4500 crRNAs constructs through nanoliter droplets organized as a microarray plate. Moreover, CARMEN-Cas13 maintains ultrasensitive SHERLOCK properties and further increases cost-effectiveness by decreasing 300-fold overall reagents costs, further demonstrating CRISPR/Cas13 as a valuable tool for early CRC diagnosis.

Recently, Cas13 systems have been further enhanced to enable a microfluidic-based system known as SATORI [193]. Although it represents a significant drop in sensitivity due to there being no isothermal amplification, it reached single-molecule resolution at approximately 5 fM. This sensitivity is obtained through a microchamber-array configuration which allows detection in less than 5 min with high specificity, positioning SATORI as a top-class, quick diagnostic tool that may also serve for CRC diagnosis and prognosis, especially for CRC-POC diagnosis efforts.

There are also reports exploiting Cas13 trans-cleavage properties for miRNAs detection (see Table 4, miRNA targets approach section). Shan et al. [179] harnessed LbuCas13 to directly detect miRNA-17, obtaining high specificity and a 1 pM sensitivity range with crRNA spacer constructs ranging from 20 nt to 28 nt in 30 min runs. Moreover, they further tested Cas13 systems specificity, evidencing its robust single-nucleotide resolution, efficiently differentiating miR-17, miR-106a, miR-20a, and miR-20b, which have 1 to 2 nt of difference, accordingly, showcasing CRISPR/Cas13-based platforms with great applicability and fidelity for multiplexed detection of highly similar miRNAs. Similar results were reported on miR-17 by Sha et al. [194], although they used a cascade CRISPR/Cas system, harnessing Cas13 and Cas14 endonucleases, obtaining a sensitivity of 1.33 fM in 15 min. However, due to the use of Cas14 as the detection molecule and Cas13 as an intermediary enzyme for the activation of a probe, multiplexation is not an option because, similarly to Cas12, Cas14 has a non-specific trans-collateral DNAse activity [159]. Another study coupled CRISPR/Cas13a to an electrochemical assay for microRNA-21 detection with a sensitivity of 2.6 fM in 60 min [195]. Nevertheless, the complete assay setup and configuration may be complex and labour intensive to construct, including the Au electrode preparation and catalytic hairpin assembly design.

It is essential to mention that there are other studies applying CRISPR/Cas13 for miRNA diagnosis, including electrochemical approaches [196] and electrochemiluminescence chip [197]. Although these systems show great results and efficiency, the configuration and construction of the assays are complex and require expert setup, reducing CRISPR/Cas13 applicability and technological accessibility for users and stakeholders. Thus, based on the available information, the most accessible systems are listed in Table 5. These methods remain quick and straightforward to setup, without compromising their overall efficiency regarding specificity and sensitivity. It is important to highlight an issue between miRNA size (18 to 24 nt) and Cas13 spacer size (20 to 28 nt). However, there are a few approaches that may solve this issue, which are mentioned in the next section.

Moreover, there is a need for research on rapid and reliable miRNA extraction methods similar to the HUDSON protocol for clinical bodily fluids such as blood and saliva. Furthermore, based on the observation of robust detection on raw clinical and environmental samples [26,187], where PCR-based configuration may not work properly due to high concentration of potential inhibitors, CRISPR/Cas13 also represents an opportunity to detect miRNA from raw or rapidly-processed samples, further facilitating sample management.

## 9. Dedicated crRNA Design for a Potential CRISPR/Cas13-Based Platform for CRC miRNAs-Based Diagnosis

To further facilitate and streamline the research and use of CRISPR/Cas13-based diagnosis and prognosis for CRC through CEx-miRNAs or cf-miRNAs, we have built a potential candidate miRNAs panel from Table 3 (best AUC reported on CRC T stage) with their respective crRNA design (see Table 5). Furthermore, several reports on miRNA detection methods, including RPA [198] and polyA/T universal tag [198,199], have not been merged with CRISPR/Cas13 for miRNA detection but represent simple approaches to solving the issue of miRNA and the corresponding spacer size.

There are two main approaches for direct CRISPR/Cas13-based CRC diagnosis (Table 5). (1) The multiplex approach, which can be obtained harnessing the di-nucleotide motif preference of each Cas13 ortholog to simultaneously detect four targets at once from a unique sample with the same detection properties [21,22]; however, only LwaCas13a is commercially available meanwhile other Cas13 endonucleases are only available as a plasmid construct. Thus, they need to be expressed and purified, which may result to be slow and expensive. (2) The singleplex approach, using only LwaCas13a, may be more achievable since numerous studies use it [21,22,185,187,192,200], showcasing a solid reproducibility.

However, these studies use intra-lab-expressed LwaCas13a and are not commercially obtained; instead, they are obtained as plasmid constructs from Addgene [201]. There are also companies selling LwaCas13a from 85 to 95% purity, similar to purity qualities obtained in previously mentioned studies. Accordingly, singleplex is currently the more affordable and promising miRNAs-based diagnosis approach due to the commercial accessibility of LwaCas13a. Meanwhile, although the multiplex approach is also promising and needed to solve CRC diagnosis challenges, using several Cas13 orthologs through plasmid expression constructs may be inconvenient due to several factors, including the need for specialized equipment and potential cross-contaminations during the overall process.

**Figure 4:**
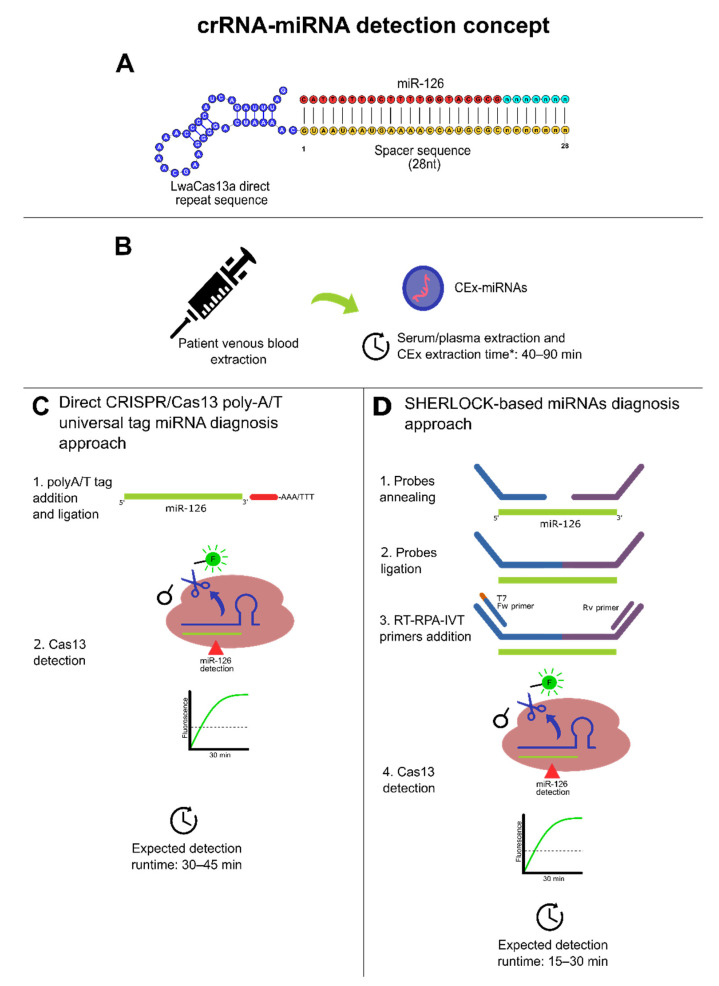
Dedicated CRISPR/Cas13-based miRNA diagnosis approaches. * Additional 30 min were added to consider miRNA extraction from exosomes. (**A**) Conceptual crRNA (direct repeat blue; spacer region: yellow; protospacer: red and cyan). (**B**) Obtention of peripheral blood from patients and CEx-miRNAs timeframe conceptualization based on [202]. (**C**) Direct CRISPR/Cas13 direct approach detection for diagnosis using a poly-A/T universal tag for CEx-miRNAs with a theoretical 2-steps method based on [178]. This approach may be suitable for highly abundant CEx-miRNAs targets. (**D**) A SHERLOCK-based diagnosis approach with a theoretical 4-steps method based on [22]. This approach may be suitable for high/low abundant CEx-miRNAs targets. For both cases (**C**,**D**), runtimes do not consider sample isolation and purification. Moreover, polyA/T ligation may need high temperatures.

Furthermore, multiplex and singleplex crRNA designs approaches can be followed using polyA/T universal tag as shown in Table 5 to comply with the spacer size of 20 to 30 nt and achieve direct Cas13 miRNAs detection in 30 to 45 min. Another potential approach can be made by coupling the RPA amplification step with annealing probes that also increase template size to comply with Cas13 crRNA spacer requirements [22]. However, Cas13 needs RNA targets, and to fulfill this requirement, RT-RPA can be followed with an in vitro transcription (IVT) to obtain the RNA template for Cas13. Thus, a SHERLOCK-based procedure may be implemented to run RT-RPA, IVT, and Cas13 reactions simultaneously (see Figure 4) to detect CRC-related miRNAs reported previously and provide results in 15 to 30 min [22] (Table 4 and Table 5). Therefore, this can be useful to expedite the diagnosis and prognosis turnaround for the medical decision-making process since SHERLOCK has a run time range from 15 to 30 min, without considering sample extraction procedures. In this regard, taking into consideration that CEx-miRNAs have shown the best results, it is pivotal to mention that the best-reported source of CEx-miRNAs from available commercial kits is blood serum, in terms of abundancy. Meanwhile, miRNA quality performed well within all used blood exosome kits extraction [202].

The difference in the turnaround time of the detection between both approaches presented in Figure 4 can be explained due to RPA, whereby it increases Cas13 targets significantly through isothermal amplification. Moreover, in terms of portability, SHERLOCK-based miRNA detection is higher as the probe ligation before RT-RPA can be performed at 37 °C, as with the rest of the process, further facilitating its configuration and platform accessibility, enabling a potential establishment of POC diagnosis and prognosis for CRC risk patients based on CRISPR-Dx technologies. Therefore, based on the presented evidence, both approaches may represent a fast route to establish a scalable, early diagnosis protocol for CRC from its cf-miRNAs/CEx-miRNAs biomarkers (e.g., miR-126, miR-1290); thus, this may set fundamental pillars for future implementations of other CRISPR/Cas13 in CRC diagnosis efforts.

## 10. Conclusions

CRC is currently a worldwide known epidemy in developed countries, whereas its prevalence and mortality are increasing yearly. To halt its advance and provide quality healthcare, there is a need to strengthen and reinforce current traditional medical surveillance programs mainly based on invasive methods such as colonoscopies or molecular methods primarily based on RT-qPCR, where both have challenges and limitations.

Recently, promising studies have revealed that CRC and other cancers actively re-lease/secrete EVs, especially CEx, which are basically intercellular communication vehicles with rich molecular cargo, including DNA, RNA, and proteins. Accordingly, CEx cargo includes miRNA, which regulates different carcinogenic physiological behaviors, including tumor progression and angiogenesis. Therein, CExs-miRNA has been reported as a potential biomarker with robust predictive results of diagnosis and prognosis.

Although the primary tool for CEx-miRNAs detection is RT-qPCR, alternative emerging molecular methods such as CRISPR-Dx technologies may potentially benefit and complement current established secondary prevention measures with cost-effective molecular early screenings. Moreover, due to the inherent ease of use, robustness, scalability and portability of CRISPR/Cas13-based detection platforms previously shown, this ultrasensitive technology may be further developed to deliver early POC diagnosis and prognosis to CRC risk patients with affordable rates and swift turnaround times, allowing healthcare teams to have reliable data to deliver the optimal treatment on time and reduce the risks of late CRC stages diagnosis which have low five-year survival rates.

Moreover, this study may also streamline future research and proof-of-concept of CRISPR/Cas13-based platforms for miRNA detection for early diagnosis and prognosis with proposed methodologies based on direct CRISPR/Cas13-miRNA detection or SHERLOCK-based miRNA detection to further enhance and facilitate CEx-miRNAs detection in minimally invasive methods, i.e., blood samples (serum) based on a CEx-miRNAs panel including miR-126, miR-1290, miR-23a, and miR-940, which have shown to date the best predictive data for early CRC stages. Thus, from the gathered evidence, CRISPR/Cas13-based platforms represent promising potential for early, next-generation CRC diagnosis and prognosis candidates, where their intrinsic features may also be appealing for the diagnosis and prognosis efforts for other types of cancers.

## Figures and Tables

**Table 1:** Current traditional clinical methods and molecular methods for CRC diagnosis.

Methods	Cost	Time *	Advantages	Disadvantages	References
Traditional methods					
Guaiac-based fecal occult blood test (gFOBT)	Low	Weeks	Biennial gFOBT screening provides sustained protection against long-term CRC mortality	Unspecific, limited sensitivity for CRC detection, requires patient dietary modification and three consecutive samples are needed.	[73,74,75,76]
Fecal immunochemical test (FIT)	Low	Weeks	Quantitative and qualitative results, user-friendly application, higher overall adherence and easier follow-up.	Test reliability decreases considerably with longer times before analysis.	[74,75,76]
Multi-target stool DNA test	High	Weeks	High sensitivity, non-invasive approach, and good benefit-risk ratios	Sensitivity is partially linked to hemoglobin thresholds and showcases a lower rate of cancer prevention	[75,77]
Colonoscopy	High	Hours	High efficacy and sensitivity on preventing CRC due to detecting and removing both advanced and non-advanced adenomas.	Invasive, need sedation and bowel cleansing. High risks linked to human manipulation errors including perforation, bleeding, and death.	[75,77]
CT colonography (virtual colonoscopy)	High	Hours	Non-invasive and effective screening test with low risk of perforation	Bowel preparation and lower sensitivity in comparison with colonoscopy.	[75,77]
Molecular methods					
qPCR	Low	Days	Minimally invasive, fast, and accurate detection. The process has been automated.	Multi-target approaches and fluorescent reporters-related applicability is variable, affecting sensitivity and specificity.	[78,79]
RT-qPCR	Low	Days	Minimally invasive and accurate detection. Currently gold-standard method.	Error-prone and reliability directly linked to sample extraction quality from clinical samples. Labor-intensive. Low portability.	[78,80]
ddPCR	Low	Days	Minimally invasive with improved analytical sensitivity to mutations such as KRAS. Reduced variability.	Trained personnel, labor-intensive, and high rates of false positives.	[81,82]
Microarrays	Medium	Days-Weeks	Minimally invasive with high sensitivity to analyze multiple targets from one sample.	Time and laborious technical procedures, along with multiple runs needed to obtain final results.	[83]
Next-generation sequencing	High	Weeks	A broader assessment of the tumor molecular profile, including mutations and ITH dynamics.	Resource-consuming and efficacy may be affected by numerous factors	[84]
CRISPR/Cas platforms	Very low	Hours-Days	Minimally invasive detection with swift, cost-effective, ultrasensitive, and specific platforms.	Detailed sequence data needed, sensitive to unidentified mutations and RNA secondary structures.	[22,24,85]

Further in-detail reading about molecular methods for CRC diagnosis can be found in [20]. * Expressed times are referential to a delivered clinical result to the patient.

**Table 2:** Most used CRC molecular biomarkers clinically used for diagnosis and prognosis.

Molecular Biomarkers	Sample Type ^a^	Example Target	Overall Effectiveness (SE/SP)	References
Adenomatous polyposis coli (APC)	Blood (DNA)	D18122V, E1317Q, and I1307K (APC polymorphisms)	NR *	[89]
Microsatellite instability (MSI)	Blood (DNA)	Bat-25, NR-21	99% (98.7/100)	[90]
Methylation (MTL)	Blood/Stool (DNA)	SEPT9	89% (90/88)	[91]
Kirsten rat sarcoma viral oncogene homolog (KRAS)	Blood (DNA)	p-21Ras mutations	60% (67/53.95)	[92]
V-raf murine sarcoma vViral oncogene homolog B1 (BRAF)	Blood (DNA)	BRAF V600 E mutation	77% (81.2/72.1)	[93]

* NR: not reported. ^a^ Overall effectiveness was calculated as ((Sensitivity + Specificity)/2); SE: sensitivity; SP: specificity. Percentage values were approximated to the nearest whole number. Further information about these and other molecular biomarkers has been reviewed in-depth elsewhere [20,60].

**Table 3:** Summary of promising reported miRNA from patient blood samples for potential CRC diagnosis and prognosis since 2017.

miRNA (By Stages) ^a^	Qualitative Regulation ^b^	AUC	Sequence (3p/5p-length-bp) ^c^	Accession Number	Qualitative Prognosis	Source	Reference
T (I & II)							
miR-126	↑	0.96	UCG UAC CGU GAG UAA UAA UGC G (3p-22)	MI0000471	Early CRC stage	CEx	[136]
miR-1290	↑	0.91	UGG AUU UUU GGA UCA GGG A (19)	MI0006352	Early CRC stage	CEx	[136]
miR-186-5p	↑	0.72	CAAA GAA UUC UCC UUU UGG GCU (21)	MI0000483	CRC early lesions	cf-miRNAs	[139]
miR-23a	↑	0.92	AUC ACA UUG CCA GGG AUU UCC (3p-21)	MI0000079	Early CRC stage	CEx	[136]
miR-423-5p	↓	0.72	UGA GGG GCA GAG AGC GAG ACU UU (23)	MI0001445	CRC early lesions	cf-miRNAs	[139]
miR-449a	↓	0.76	UGG CAG UGU AUU GUU AGC UGG U (22)	MI0001648	Poor prognosis, lower overall survival	cf-miRNAs	[140]
miR-592	↑	0.80	UUG UGU CAA UAU GCG AUG AUG U (22)	MI0003604	Early CRC stage	cf-miRNAs	[141]
miR-940	↑	0.90	AAG GCA GGG CCC CCG CUC CCC (21)	MI0005762	Early CRC stage	CEx	[136]
N (III)							
miR-1539	↑	0.67	UCC UGC GCG UCC CAG AUG CCC (21)	MI0007260	CRC lymph node invasion and poor clinicopathological behavior	CEx	[142]
miR-19a	↑	0.87	UGU GCA AAU CUA UGC AAA ACU GA (3p-23)	MI0000073	CRC invasion	cf-miRNAs	[143]
miR-20a	↑	0.83	UAA AGU GCU UAU AGU GCA GGU AG (5p-23)	MI0000076	CRC increasing distant metastasis rates	cf-miRNAs	[143]
miR-150	↑	0.75	UCU CCC AAC CCU UGU ACC AGU G (5p-22)	MI0000479	CRC promoting epithelial to mesenchymal transition	cf-miRNAs	[143]
miR-552	↑	NR	AAC AGG UGA CUG GUU AGA CAA (3p-21)	MI0003557	CRC poor prognosis, worse 5-year overall survival	cf-miRNAs	[144]
M (IV)							
miR-126-3p	↑	NR	UCG UAC CGU GAG UAA UAA UGC G (22)	MI0000471	*Progression-free survival	cf-miRNAs	[145]
miR-155-5p	↑	NR	UUA AUG CUA AUC GUG AUA GGG GUU (24)	MI0000681	*Short progression-free survival	cf-miRNAs	[146]
miR-17-5p	↑	0.90	CAA AGU GCU UAC AGU GCA GGU AG (23)	MI0000071	CRC increased invasive ability and metastasis potential	CEx	[147]
miR-19b	↑	0.89	UGU GCA AAU CCA UGC AAA ACU GA (3p 23)	MI0000074	High amounts indicate metastatic CRC	CEx	[132]
miR-20b-5p	↑	NR	CAA AGU GCU CAU AGU GCA GGU AG (23)	MI0001519	*Progression-free survival	cf-miRNAs	[146]
miR-21	↑	0.98	UAG CUU AUC AGA CUG AUG UUG A (5p-22)	MI0000077	High amounts indicate metastatic CRC	CEx	[132]
miR-222	↑	0.90	AGC UAC AUC UGG CUA CUG GGU (3p-22)	MI0000299	Higher amounts indicate a lower overall survival rate	CEx	[132]
miR-29b-3p	↑	NR	UAG CAC CAU UUG AAA UCA GUG UU (23)	MI0000105	*Progression-free survival	cf-miRNAs	[146]
miR-320d	↑	0.63	AAA AGC UGG GUU GAG AGG A (19)	MI0008190	Distinguish metastatic from non-metastatic CRC.	CEx	[148]
miR-92a	↑	0.95	UAU UGC ACU UGU CCC GGC CUG U (3p-22)	MI0000093	Higher amounts indicate a higher risk of tumor progression	CEx	[132]
miR-92a-3p	↑	0.85	UAU UGC ACU UGU CCC GGC CUG U (22)	MI0000093	CRC increased invasive ability and metastasis potential	CEx	[147]

NR: not reported; ↑: upregulated; ↓: downregulated. * miRNAs associated with a prognosis after treatment were delivered. ^a^ Mentioned miRNAs are often present during all the stages of the disease, although their expression level shifts and are significant compared to healthy controls. ^b^ In comparison with healthy controls. There can be variations depending on the CRC mutation type. ^c^ Stem-loop sequences were not considered. Reference sequences were obtained from [149,150]. When 3p/5p were not mentioned, the annotation with higher reads was used when possible. A focused review on circulating exosomal miRNA and their role in the diagnosis, prognosis, surveillance, and monitoring of CRC can be found in [138].

**Table 4:** Promising molecular CRISPR/Cas13-based platforms for CRC early diagnosis and prognosis showcasing potential applicability and technological accessibility.

Methods ^a^	Target	Pre-Amplification (Method)	Sensitivity ^b^	Runtime (min)	Multiplexation	Readout	Reference
miRNA targets approach							
CRISPR/LbuCas13a	miRNAs	N	5 pM	30	N	F	[179]
non-miRNA targets approach							
SHERLOCK	ST	Y (RPA)	2 aM	120	N	F	[21]
SHERLOCKv2	ST	Y (RPA)	8 zM	30	Y (4)	F/S	[22]
HUDSON	ST	Y (RPA)	0.9 aM	120	N	F/S	[26]
CARMEN-Cas13	Viral particles	Y (PCR)	2 aM	30–180	Y (169)	F	[192]
SATORI	SARS-CoV-2	N	5 fM	5–10	N	F	[193]

Y = Yes; N = No; F = fluorescence; S = lateral flow strip; ST = synthetic target. SHERLOCK = specific high sensitivity enzymatic reporter unlocking; HUDSON = heating unextracted diagnostic samples to obliterate nucleases; CARMEN = combinatorial arrayed reactions for multiplexed evaluation of nucleic acids; SATORI = CRISPR-based amplification-free digital RNA detection. ^a^ All methods reported single nucleotide, with the exception of SATORI. bfM = 10–15 mol L-1; aM = 10–18 mol L-1; zM = 10–21 mol L-1. An in-depth review of the CRIPSR/Cas13 nucleic acid detection tool and other Cas endonucleases can be found in [85].

**Table 5:** CRC CEx-miRNAs panels for direct multiplex and singleplex approaches of CRISPR/Cas13-based diagnosis.

miRNA	crRNA Sequence ^a^	Cas13 Ortholog (Spacer Length)	Associated FQR	Reference
Multiplex approach ^b^				
miR-126-3p	{GAU UUA GAC UAC CCC AAA AAC GAA GGG GAC UAA AAC}–[AGC AUG GCA CUC AUU AUU ACG C (uuu uuu)]	LwaCas13a (28 nt)	F//T*A*rArUG*C//Q	[24,136,197]
miR-1290	{GUU GAU GAG AAG AGC CCA AGA UAG AGG GCA AUA AC}–[ACC UAA AAA CCU AGU CCC U (uuu uuu uuu)]	LbaCas13a (28 nt)	F//T*A*rUrAC*C*//Q	[24,136,197]
miR-23a-3p	[UAG UGU AAC GGU CCC UAA AGG (uuu uuu uuu)]–{GUU GUA GAA GCU UAU CGU UUG GAU AGG UAU GAC AAC}	CcaCas13b (30 nt)	F//T*A*rUrAG*C*//Q	[24,136,197]
miR-940	[UUC CGU CCC GGG GGC GAG GGG (uuu uuu uuu)]–{GUU GUA GAA GCU UAU CGU UUG GAU AGG UAU GAC AAC}	PsmCas13b (30 nt)	F//rArArArArA//Q	[24,136,197]
Singleplex approach ^c^				
miR-126-3p	{GAU UUA GAC UAC CCC AAA AAC GAA GGG GAC UAA AAC}–[AGC AUG GCA CUC AUU AUU ACG C (uuu uuu)]	LwaCas13a (28 nt)	F//T*A*rArUG*C//Q	[24,136,197]
miR-1290	{GAU UUA GAC UAC CCC AAA AAC GAA GGG GAC UAA AAC}–[ACC UAA AAA CCU AGU CCC U (uuu uuu uuu)]	LwaCas13a (28 nt)	F//T*A*rArUG*C//Q	[24,136,197]
miR-23a-3p	{GAU UUA GAC UAC CCC AAA AAC GAA GGG GAC UAA AAC}–[UAG UGU AAC GGU CCC UAA AGG (uuu uuu u)]	LwaCas13a (28 nt)	F//T*A*rArUG*C//Q	[24,136,197]
miR-940	{GAU UUA GAC UAC CCC AAA AAC GAA GGG GAC UAA AAC}–[UUC CGU CCC GGG GGC GAG GGG (uuu uuu u)]	LwaCas13a (28 nt)	F//T*A*rArUG*C//Q	[24,136,197]

F = fluorophore; nt = nucleotides; Q = quencher; r = ribonucleotide; Lba = *Lachnospiraceae bacterium* NK4A179; Cca = *Capnocytophaga canimorsus* Cc5; Psm = *Prevotella* sp. MA2016; T*, A*, C* or G* are phosphorothioate modifications. ^a^ Sequences between {} are the direct repeat sequences from each Cas13 endonuclease ortholog. Sequences between [] are spacer regions complementary to the miRNA sequence accession number given in Table 3 that have been ligated to a poly A/T universal tag at their 3′ end denoted between () to increase target length towards 28 or 30 nt according to the Cas endonuclease proposed. Note that the spacer sequence is complementary to the target miRNAs. Where miR-3p/5p was not mentioned, we have used the 3p/5p with higher reads as a standard annotation for the proposed designs. ^b^ Multiplex approach. Simultaneous detections from a single sample in a single tube. ^c^ Singleplex approach. Simultaneous detections from a single sample in multiple tubes.

## Data Availability

No new data were created or analyzed in this study. Data sharing is not applicable to this article.
